# Middle–Late Jurassic fossils from northeastern China reveal morphological stasis in the catkin-yew

**DOI:** 10.1093/nsr/nwaa138

**Published:** 2020-06-18

**Authors:** Chong Dong, Gongle Shi, Fabiany Herrera, Yongdong Wang, Patrick S Herendeen, Peter R Crane

**Affiliations:** State Key Laboratory of Palaeobiology and Stratigraphy, Nanjing Institute of Geology and Palaeontology and Center for Excellence in Life and Paleoenvironment, Chinese Academy of Sciences, China; State Key Laboratory of Palaeobiology and Stratigraphy, Nanjing Institute of Geology and Palaeontology and Center for Excellence in Life and Paleoenvironment, Chinese Academy of Sciences, China; Chicago Botanic Garden, USA; State Key Laboratory of Palaeobiology and Stratigraphy, Nanjing Institute of Geology and Palaeontology and Center for Excellence in Life and Paleoenvironment, Chinese Academy of Sciences, China; Chicago Botanic Garden, USA; Oak Spring Garden Foundation, USA and; School of Forestry and Environmental Studies, Yale University, USA

The Taxaceae, commonly known as the yews, are widely used in ornamental horticulture and are an important source of chemotherapeutic drugs (e.g. Paclitaxel). The Taxaceae have long been considered distinct from other conifers, based on their axillary seed-bearing structures that are very different from most conifer cones, consisting of an axis bearing a single terminal seed with a fleshy aril, subtended by several bracts [1].

Phylogenetic analyses based on molecular data recognize the Taxaceae *sensu stricto* as monophyletic comprising five genera [2]. *Amentotaxus* and *Torreya* are sister taxa, and together are sister to the Taxeae, which comprises *Taxus*, *Pseudotaxus* and *Austrotaxus. Cephalotaxus* is resolved as sister to Taxaceae *sensu stricto*. The Taxaceae *sensu stricto* comprise about 25 extant species, many of which have very restricted distributions and small population sizes, and are endangered in the wild [3]. Fossil evidence of Taxaceae is based mainly on isolated leaves or leafy shoots for which the reproductive structures are unknown. However, several more complete fossils with attached seed-bearing structures show that Taxaceae had diverged from other conifers by the earliest Jurassic and were probably diverse during the Jurassic and Cretaceous [4,5].

Here we add to knowledge of early Taxaceae based on fossils from the late Middle–early Late Jurassic Daohugou Bed (∼165–158 Ma) in eastern Inner Mongolia, northeastern China [6]. The material includes the terminal portion of a leafy shoot with attached seed-bearing structures (Fig. 1a and e), and a leafy shoot with two orders of branching in which each ultimate shoot has a terminal conical bud (Fig. 1b and f). These fossils (for detailed description and illustration see Supplementary Data) bear a striking resemblance to the leafy shoots and seed-bearing structures of extant *Amentotaxus* (Fig. 1c, d and i).

As in extant *Amentotaxus*, the leafy shoots in the Daohugou fossils spread more or less in a single plane; the leaves are linear-lanceolate and appear to be borne in two ranks, although they are decussately inserted (Fig. 1b and f). The leaves also have a raised mid-vein on the adaxial surface and two broad lateral stomatal bands on the abaxial surface. In the seed-bearing specimen from Daohugou, and also in extant *Amentotaxus*, each seed is borne singly and erect on a short, naked axis that arises in the axil of a vegetative leaf (Fig. 1g, h and j, and Supplementary Fig. 4). The seed is not visible in the Daohugou fossil, but this is often also the case in extant *Amentotaxus*, where the small, immature seeds are often completely, or almost completely, enclosed by the opposite and decussate bracts (Fig. 1i). In all the features that are preserved, the fossil material only differs from extant *Amentotaxus* in having shorter seed-bearing axes.

**Figure 1.**
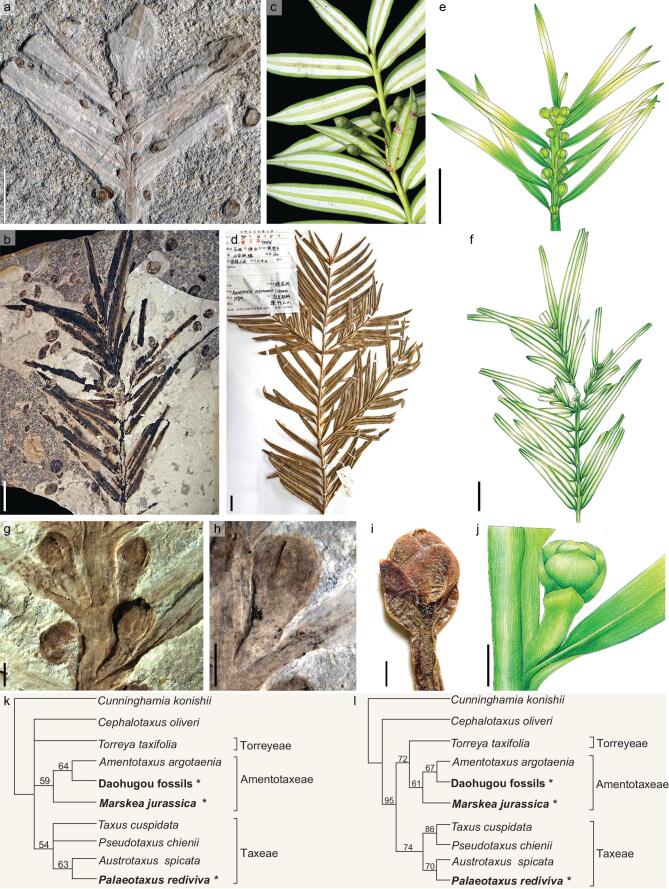
Fossil cf. *Amentotaxus*, extant *Amentotaxus*, and phylogenetic relationships of the fossils. (a), (b), (e), (f), (g), (h) and (j) cf. *Amentotaxus* from the Middle–Late Jurassic Daoguhou Bed in eastern Inner Mongolia, China. (a) Ultimate leafy shoot with attached axillary seed-bearing structures. B0498a (Institute of Vertebrate Paleontology and Paleoanthropology, CAS). (b) Leafy shoot of two orders spreading more or less in a single plane, with oppositely arranged ultimate shoots. PB23120a (Nanjing Institute of Geology and Palaeontology, CAS). (e) Reconstructive drawing of specimen in (a) based on the part, counterpart and micro-CT volume rendering. (f) Line drawing of the two-order leafy shoot in (b). (g) Middle region of ultimate leafy shoot showing decussate leaves, each subtending a single seed-bearing structure. Counterpart of the specimen in (a). B0498b. (h) Detail of seed-bearing structure showing the short naked axis and decussate bracts subtending the seed. B0498b. (j) Line drawing of individual seed-bearing structure based on (h) showing the short naked axis and decussate bracts subtending and enclosing the terminal seed (see also Supplementary Data). (c), (d) and (i) Extant material of *Amentotaxus* for comparison. (c) *A. yunnanensis*, leafy shoot with attached, immature seed-bearing structures. (d) *A. argotaenia*, leafy shoot showing opposite branching. Herbarium specimen. KUN No. 0763276. (i) *A. argotaenia*, detail of seed-bearing structure showing immature seed nearly completely enclosed by subtending bracts. Herbarium specimen. NAS00165854. (k) and (l) Phylogenetic analyses of selected species of extant and fossil Taxaceae (Supplementary Data). Fossil species in bold type and indicated with a star; numbers at nodes indicate bootstrap support values. (k) Strict consensus of 14 most parsimonious trees (25 steps, CI = 0.72, RI = 0.65) from an unconstrained parsimony analysis. (l) Single most parsimonious tree (26 steps, CI = 0.69, RI = 0.60) from molecular scaffold analysis with relationships of extant species fixed to a backbone based on molecular data [2]. Scale bars: (a), (b), (e), (f) = 1 cm; (d) = 2 cm. (g), (h), (i), (j) = 1 mm.

All other extant genera of Taxaceae differ from the Daohugou fossils. *Torreya* has opposite to sub-opposite leaves, but its seed-bearing structures lack a distinct stalk. *Austrotaxus*, *Pseudotaxus* and *Taxus* have seed-bearing structures that arise singly from the axil of a normal vegetative leaf as in the Daohugou fossils, but their leaves are helically arranged and the seed-bearing stalks are very short and covered by dense scale leaves.

A close relationship of the fossil material to extant *Amentotaxus* is also supported by phylogenetic analyses based on morphological characters of living and fossil Taxaceae (Supplementary Data). In an unconstrained analysis (Fig. 1k), and also an analysis with the relationships of extant Taxaceae constrained to the topology of the most recent molecular phylogeny (Fig. 1l), the Daohuguo fossils are resolved as sister taxa to extant *Amentotaxus*, with *Marskea jurassica* from the Middle Jurassic of Yorkshire, England [4,5] sister to both. The clade comprising *Amentotaxus*, the Daohuguo fossils and *M. jurassica* is defined by the naked seed stalks that each bear a single seed. It would therefore be reasonable to assign the Daohuguo fossils to the extant genus *Amentotaxus*, while also recognizing that there is currently insufficient information on pollen organs and other structures that could place it unequivocally in the crown group of the genus.

The morphological distinctiveness of Taxaceae has been used to suggest they are a separate line of evolution from other conifers [1], but evidence from molecular phylogenetics [2], as well as other assessments [7], include Taxaceae *sensu stricto* plus *Cephalotaxus* among other conifers as the sister group to Cupressaceae *sensu lato* (Fig. 1l). The earliest reliable record of Taxaceae is *Palaeotaxus rediviva* from the Early Jurassic (Hettangian) of southern Sweden [4].

In addition to the Daohugou fossils, *M*. *jurassica* and *P*. *rediviva*, there are also several detached seeds, seed-bearing axes and leafy shoots known from the Jurassic and Cretaceous. Together, these fossils clearly document that the lineage including the five extant genera had diverged from other conifers by at least the Early Jurassic and that the Taxaceae were diverse during the later Mesozoic. Recognition of *Amentotaxus* in the Daohugou biota, together with *M*. *jurassica*, indicates that differentiation of the Amentotaxeae was already underway by the Middle Jurassic.

China is rich in plant species that were once more widely distributed across the Northern Hemisphere [8]. In most cases, the credible fossil record of relictual genera such as *Craigia* (Malvaceae), *Davidia* (Nyssaceae), *Cyclocarya* (Juglandaceae) and *Metasequoia* (Cupressaceae) extends back to the early Cenozoic [8]. In a few other cases, the reliable fossil record of conifers such as *Taiwania* (Cupressaceae) and several angiosperm genera, such as *Hedyosmum* (Chloranthaceae) [9], extends back into the Cretaceous. However, there are few living genera of seed plants for which the fossil record extends back into the Jurassic, and still fewer that are well documented based on details of both leaves and reproductive structures. The most comparable case to *Amentotaxus* is *Ginkgo*, which is well documented as *Ginkgo yimaensis* from the Middle Jurassic of Henan, central China [10]. Just as the seed-bearing structures of Daohugou fossils differ slightly from those of extant *Amentotaxus* species, the seed-bearing structures of *G*. *yimaensis* differ from those of extant *Ginkgo biloba* [10]. Nevertheless, as in *Ginkgo*, the extent of structural stasis between Daohugou fossils and extant species of *Amentotaxus* is striking, and highlights *Amentotaxus* as a living fossil like *Ginkgo* that has undergone little morphological change since the Middle–Late Jurassic.

## Supplementary Material

nwaa138_Supplemental_File
