# Supplementary material for: Middle–Late Jurassic fossils from northeastern China reveal morphological stasis in the catkin-yew
Source: Natl Sci Rev. 2020 Jun 18;7(11):1765–7. doi: 10.1093/nsr/nwaa138 (PMC8288717; doi:10.1093/nsr/nwaa138)
Supplement: nwaa138_Supplemental_File [file nwaa138_supplemental_file.pdf]

Supplementary Data

Middle–Late Jurassic fossils from northeastern China reveal morphological stasis in the catkin-yew

Chong Dong [1], Gongle Shi [1], Fabiany Herrera [2], Yongdong Wang [1], Patrick S. Herendeen [2], Peter R. Crane [3, 4]

[1] State Key Laboratory of Palaeobiology and Stratigraphy, Nanjing Institute of Geology and Palaeontology and Center for Excellence in Life and Paleoenvironment, Chinese Academy of Sciences, Nanjing 210008, China;

[2] Chicago Botanic Garden, Glencoe, IL 60022, USA;

[3] Oak Spring Garden Foundation, Oak Spring, Upperville, VA 20184, USA;

[4] School of Forestry and Environmental Studies, Yale University, New Haven, CT 06511, USA.

Supplementary data include the following files:

Material and methods

Supplementary Figure 1

Supplementary Figure 2

Supplementary Figure 3

Supplementary Figure 4

Supplementary Figure 5

Supplementary Figure 6

Description of Fossil Material

Comparison of the Daohugou fossils with fossil Taxaceae

Characters and character scorings

Supplementary Table 1

References

Material and Methods

Geological setting and age

The fossil specimens were collected from the Daohugou Bed in Daohugou Village (41°19′25″N, 119°14′40″E; Fig. S1) in Ningcheng County, eastern Inner Mongolia, northeastern China. The Daohugou Bed within the Haifanggou Formation, comprises a thick sequence of greyish to green, very finely laminated tuffaceous silty claystones alternating with layers of tuff (Huang 2016). It is well-known for yielding diverse, exceptionally well-preserved vertebrate (Huang 2016; Xu et al. 2016), invertebrate (Huang 2016; Xu et al. 2016), and plant fossils (Dong et al. 2016; Na et al. 2017; Pott and Jiang 2017). The Daohugou fossil plant assemblage includes algae, bryophytes, Lycopodiaceae, sphenophytes, Filicales, Bennettitales, Coniferales, Ginkgoales, Czekanowskiales, Caytoniales, and some seeds and cones of uncertain affinity. The occurrence of *Amentotaxus* in the Daohugou Bed was briefly reported in the preliminary account of the Daohugou plant fossil assemblage (Dong et al. 2016). Radiometric dating of the volcanic rocks overlying the fossil bed constrains the age of the Daohugou Biota as not younger than late Middle–early Late Jurassic (~165–158 Ma) (Huang 2016; Xu et al. 2016).

Fossil repository, preparation and photography

The fossil material consists of two specimens. Specimen B0498a, b (Figs. 1a, S2b) was collected in the Daohugou Village by the western Liaoning Expedition team of the Institute of Vertebrate Paleontology and Paleoanthropology, CAS during field work in 2003, and is deposited in the Institute of Vertebrate Paleontology and Paleoanthropology, CAS in Beijing, China. Specimen PB23120a, b (Figs. 1b, S2a) was also collected in Daohugou Village by a local farmer in the Spring of 2017 and is deposited in the Nanjing Institute of Geology and Palaeontology, CAS in Nanjing, China.

Specimens were photographed using a Sony α6000 digital camera with a Sony SEL30M35 microlens. Details of leaf surface, terminal buds, and seed-bearing structures were photographed using a Lecia M205A stereomicroscope equipped with a Leica DFC450 digital camera. Cuticle fragments were obtained from leaf compressions of specimen PB23120a by maceration with Schultze's solution (35% HNO_3 with a few crystals of $KClO_3$). Isolated cuticle fragments were mounted on stubs, coated with gold, and examined and

photographed using a Leo1530 VP field-emission scanning electron microscope (SEM) at the Nanjing Institute of Geology and Palaeontology, CAS. Selected leaf areas of the impression fossil were cleaned gently with dilute hydrochloric acid (HCL), and examined directly with SEM without coating. Three-dimensional morphology was examined using a General Electric dual-tube X-ray computed tomography scanner in the Department of Organismal Biology and Anatomy, University of Chicago. Scans were performed with a target at 220 μA and 120 kV, 500 ms exposure time for 1700 projections. Datasets were processed using Avizo 8.1 and Meshlab (2016.12) to obtain successive virtual sections, translucent volumes, and surface renderings.

Phylogenetic analysis

The cladistic analysis used a matrix of 13 morphological characters for six extant species (as placeholders for the five extant genera of Taxaceae *sensu stricto* and *Cephalotaxus*), as well as the Daohugou fossils and the Jurassic *Marskea jurassica* and *Palaeotaxus rediviva*. *Cephalotaxus* was included in the analysis because phylogenetic studies based on molecular data suggest that it is sister to Taxaceae *sensu stricto* (Cheng et al. 2000; Ran et al. 2018). Analyses were performed with and without a backbone constraint provided by results from phylogenetic analyses of extant conifers using molecular data (Cheng et al. 2000; Ran et al. 2018). *Cunninghamia konishii* Hayata was chosen as the outgroup because it is an early diverging member of the Cupressaceae *sensu lato* (Mao et al. 2012; Shi et al. 2014), which are resolved as the sister group to Taxaceae *sensu stricto* + *Cephalotaxus* (Leslie et al. 2012; Ran et al. 2018). Parsimony analyses were performed using a heuristic search in PAUP*, version 4.0a167, with tree-bisection-reconnection branch swapping, random addition sequence, 1,000 replicates, and MulTrees on. All characters were treated as unordered and weighted equally. Tree support was assessed using bootstrap analyses with 10,000 bootstrap replicates and heuristic search with 10 random sequence additions. Changes of morphological characters on the tree were traced using Mesquite, version 3.61 (www.mesquiteproject.org).

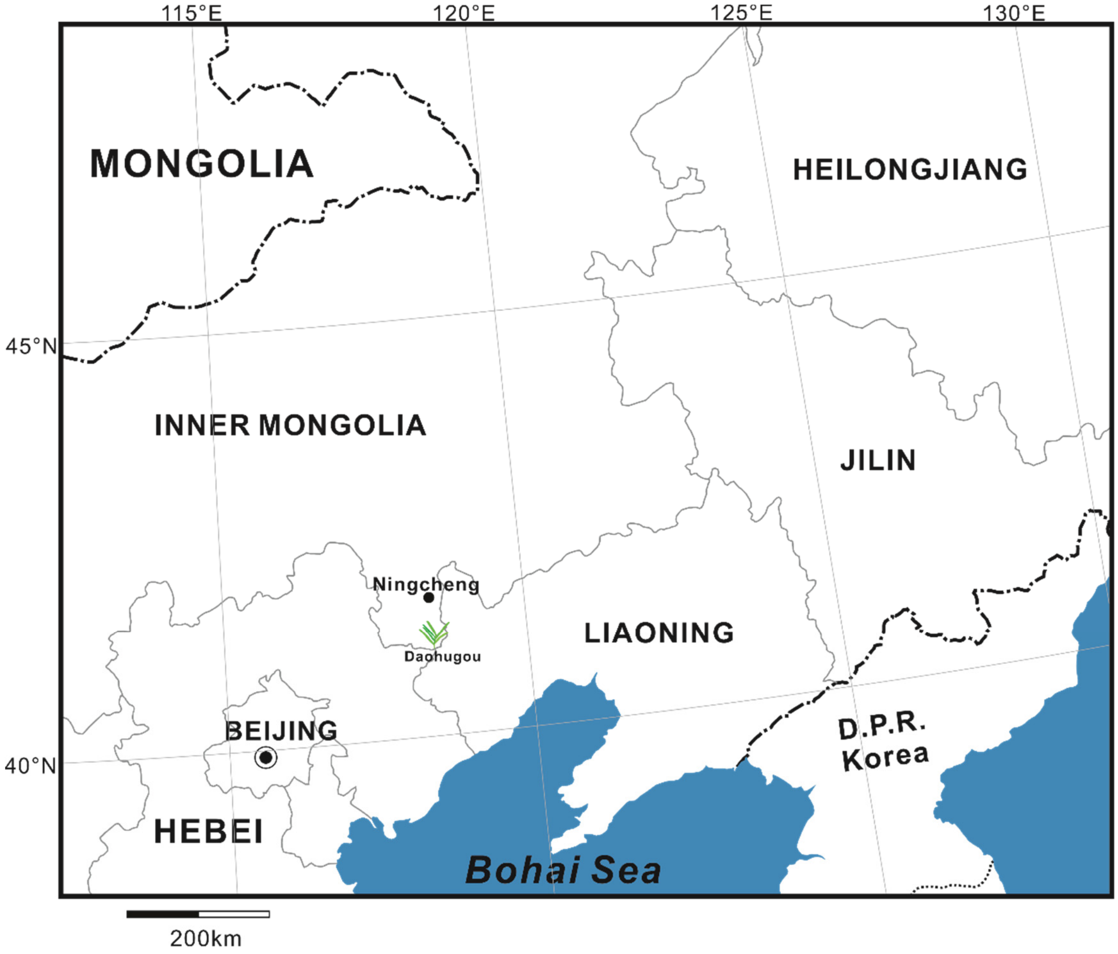

Supplementary Figure 1. Map showing location of the Daohugou locality in eastern Inner Mongolia, China.

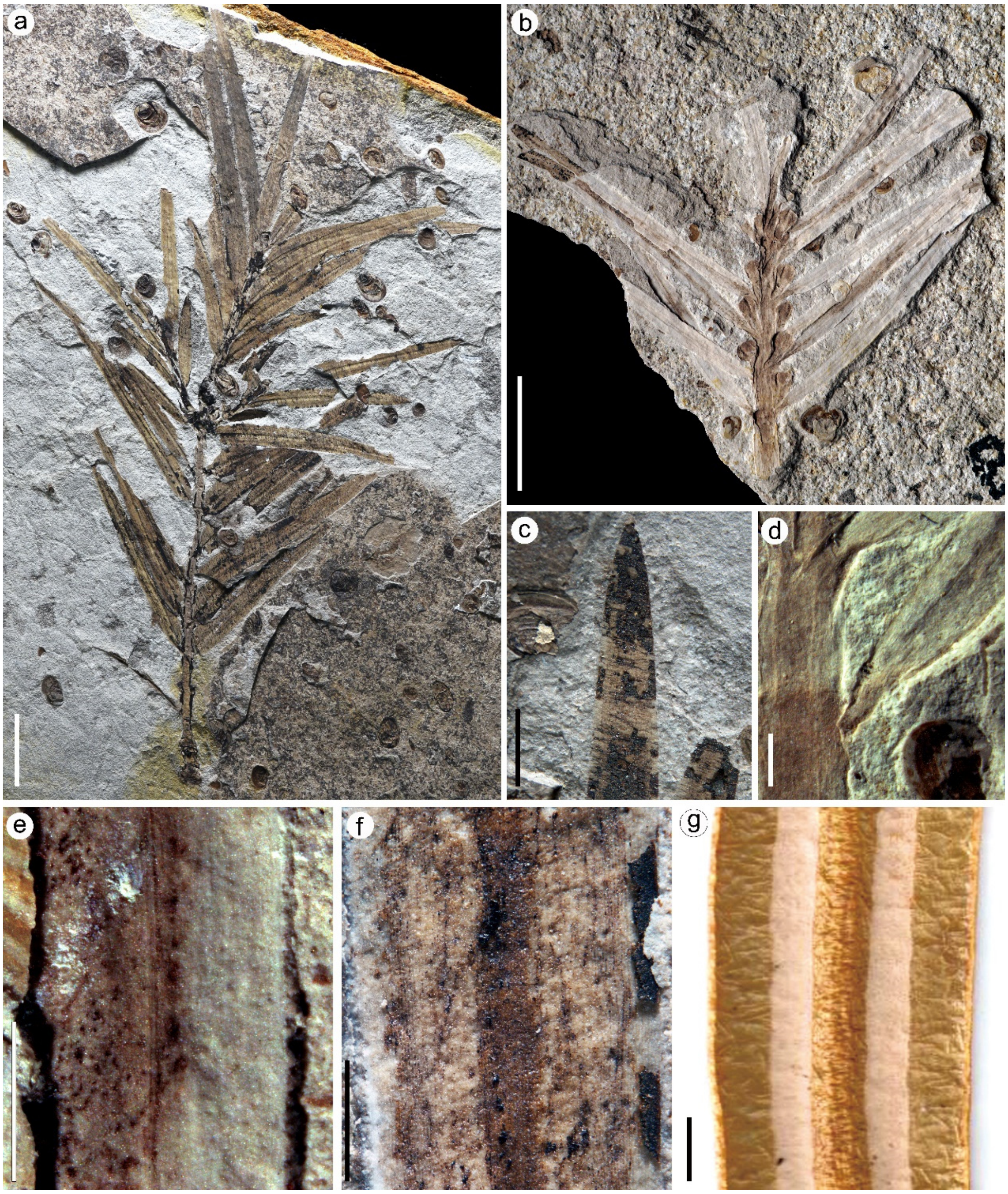

Supplementary Figure 2. (a–f) cf. *Amentotaxus* from the Middle–Late Jurassic Daoguhou Bed in eastern Inner Mongolia, China, light micrographs. (a) Leafy shoot of two orders spreading more or less in a single plane. Counterpart of specimen in Fig. 1b (main text). PB23120b. (b) Ultimate leafy shoot with attached axillary seed-bearing structures. Counterpart of specimen in Fig. 1a (main text). B0498b. (c) Upper portion of leaf showing acute apex, enlarged from Fig. 1b (main text). PB23120a. (d) Lower region of leaf showing constricted and decurrent leaf base, enlarged from Fig. 1a (main text). B0498a. (e) Adaxial leaf surface showing impression of raised midvein, enlarged from (b). B0498b. (f) Abaxial leaf surface showing two broad stomatal bands and narrow marginal zone, enlarged from Fig. 1b (main text). PB23120a. (g) Extant *Amentotaxus argotaenia*, abaxial leaf surface showing two distinct stomatal bands and broad marginal zone. Scale bars: a, b = 10 mm; c–g = 1 mm.

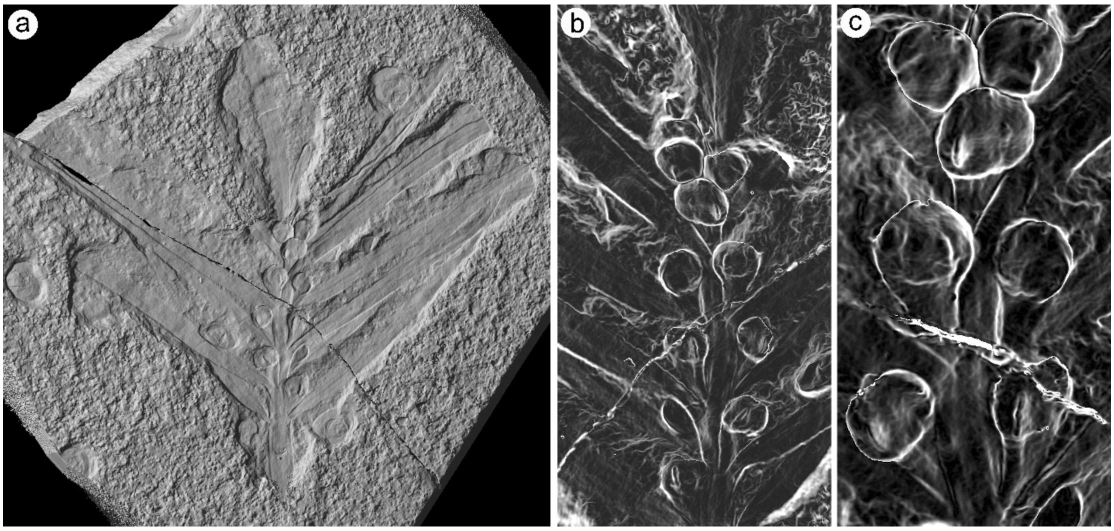

Supplementary Figure 3. cf. *Amentotaxus* from the Middle–Late Jurassic Daoguhou Bed in eastern Inner Mongolia, China, micro-CT volume renderings. B0498a. (a) isosurface rendering. (b, c) Detail of oppositely arranged seed-bearing structures that singly arise from the axils of normal vegetative leaves.

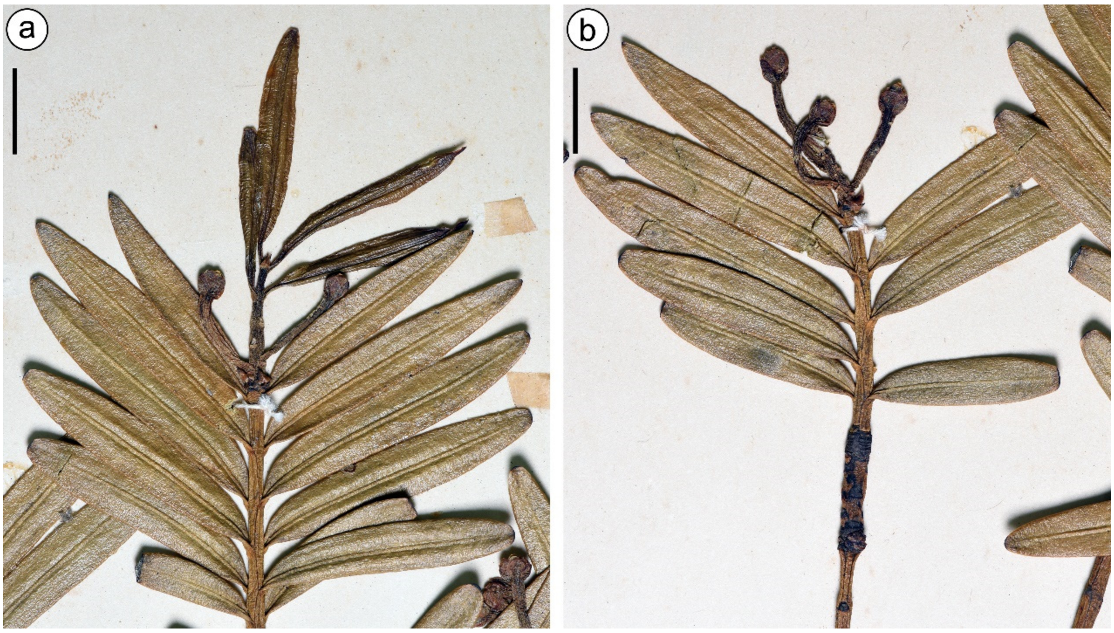

Supplementary Figure 4. (a, b) *Amentotaxus argotaenia*, extant ultimate leafy shoot with attached seed-bearing structures. Herbarium specimen. NAS00165854. Scale bars: a, b = 10 mm.

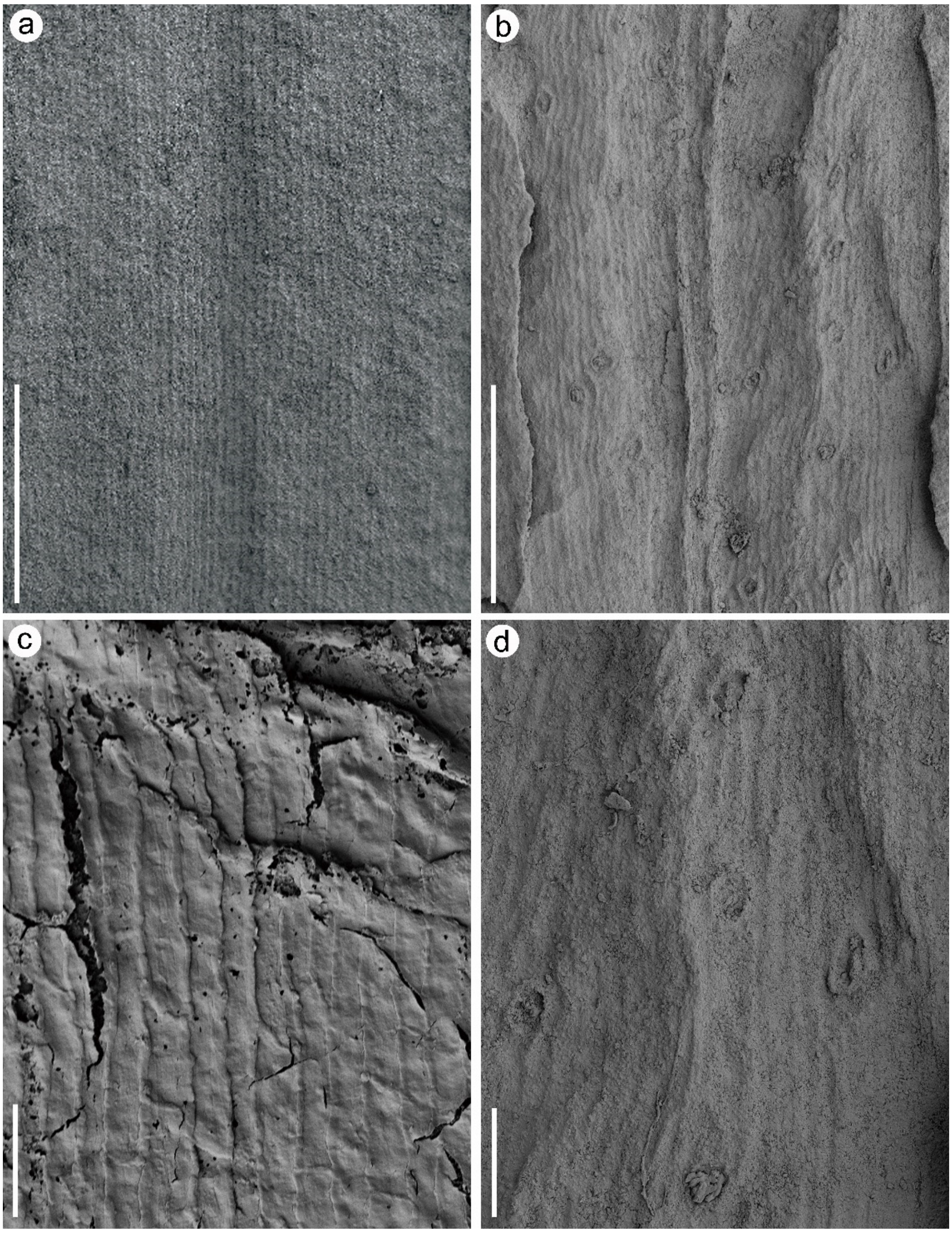

Supplementary Figure 5. cf. *Amentotaxus* from the Middle–Late Jurassic Daoguhou Bed in eastern Inner Mongolia, China, scanning electron micrographs. (a) Impression of adaxial leaf surface showing the median groove resulting from the raised midvein, and rectangular epidermal cell outlines arranged in longitudinal files. B0498b. (b) Impression of abaxial leaf surface showing impression of midrib and two broad lateral stomatal bands with sparse scattered stomata. B0498b. (c) Inner surface of abaxial cuticle over the midrib, showing rectangular epidermal cell outlines arranged in longitudinal files. PB23120a. (d) Impression of abaxial leaf surface showing detail of sparse stomata. B0498b. Scale bars: a, b = 500 μm; c = 50 μm; d = 100 μm.

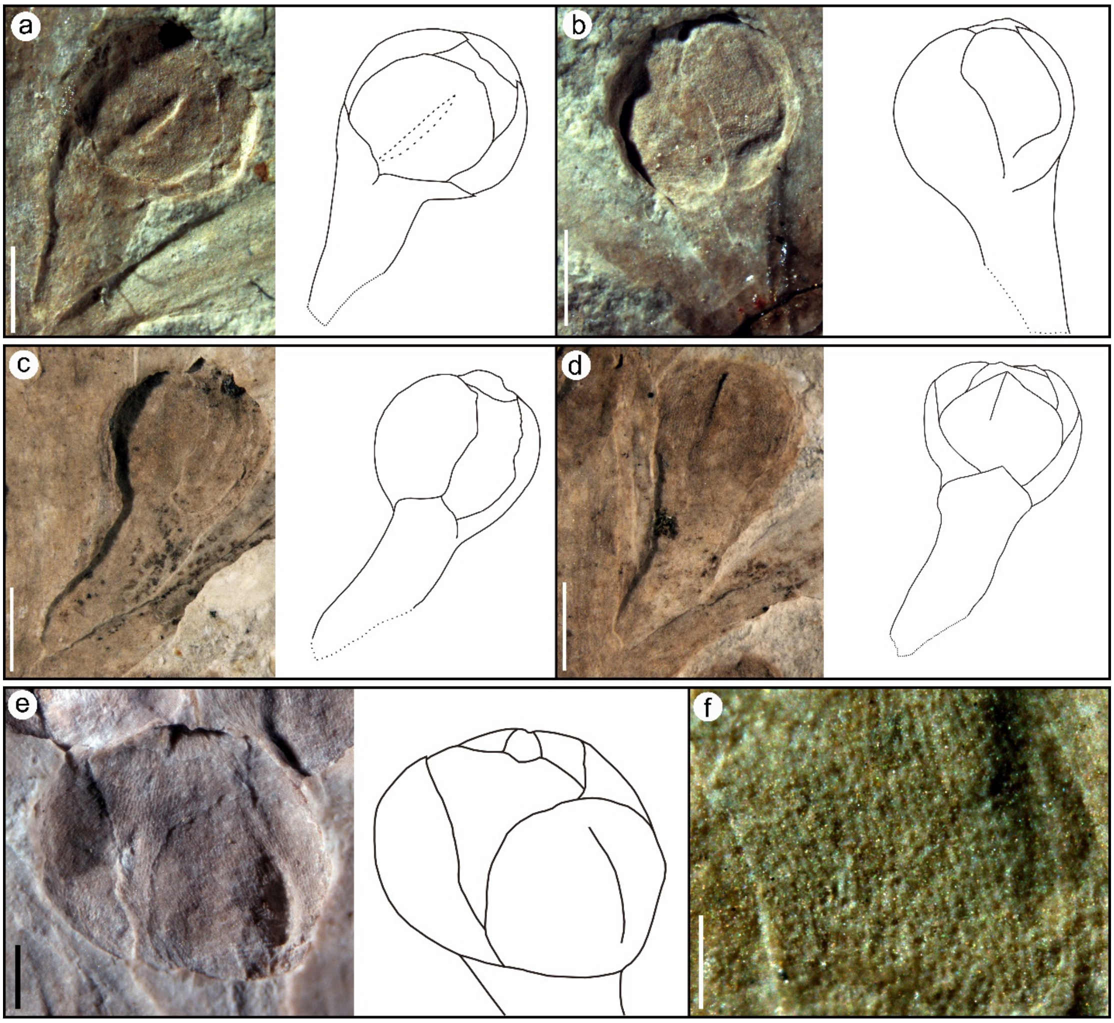

Supplementary Figure 6. (a–f) cf. *Amentotaxus* from the Middle–Late Jurassic Daoguhou Bed in eastern Inner Mongolia, China. (a–e) Details of seed-bearing structures, with interpretative line drawings, showing decussate bracts subtending and enclosing the terminal seed. (a, b, d, e) Enlarged from Fig. 1a (main text). B0498a. (c) Enlarged from Fig. S1b. B0498b. (f) Detail of outer surface of bract, showing epidermal cell outlines. B0498b. Scale bars: a–d = 1 mm; e = 500 μm; f= 200 μm.

Description of Fossil Material

We do not establish a new species for the studied Daohugou fossils because of space constraints in the main body of the paper. A detailed description of the fossils is provided below and a short note and diagnosis for the new species will be published elsewhere.

The fossil material consists of two specimens, including an impression of an ultimate leafy shoot with attached seed-bearing structures (B0498a, b: Figs. 1a, S2b) and a compression of a sterile branching system of two orders (PB23120a, b: Figs. 1b, S2a). The specimens are considered to belong to the same species based on similarities in the form, size, and arrangement of the leaves.

Branches of the leafy shoot are opposite, and spread more or less in a single plane (Figs. 1b, S2a). Penultimate leafy shoots have a subterete axis 1.2–1.7 mm thick and are up to 60 mm wide across the two ranks of leaves. Axes of the ultimate leafy shoots are ca. 0.6–1.0 mm thick, ca. 12–26 mm long, and spread at angles of 34°–40° from the axis of the penultimate shoot (Figs. 1b, S2a). Each ultimate shoot is terminated by an ovoid conical bud (Figs. 1b, S2a), 1.2–2.2 mm long and 1.1–1.8 mm wide.

Insertion of the leaves is opposite and decussate, but they appear in two ranks due to twisting of their constricted leaf bases (Fig. 1g). Leaves are spaced ca. 3–4 mm apart, spread at angles of ca. 32°–45° from the shoot axis, and are often partially overlapping (Fig. 1a, 1b).

Leaves are linear-lanceolate with entire margins, and are straight or slightly curved away from the axis on which they are borne (Fig. 1a, 1b). Leaves of the penultimate leafy shoot are typically 45–55 mm long, 4.0–4.2 mm wide, while leaves of the ultimate leafy shoots are 32–35 mm long, and up to 2.8 mm wide. Leaves are sessile and constricted into a narrow, decurrent, stalk-like, base (Figs. 1g, S2d). The widest point of the leaf is slightly below the midpoint, from which the leaf tapers gradually towards the acute leaf apex (Fig. S2c).

Leaves are dorsiventrally flattened, single-veined, and with the midvein slightly raised on the adaxial surface, which lacks stomata (Fig. S2e). The midvein is not raised on the abaxial surface but is nonetheless prominent and is flanked by two broad stomatal bands 250–400 μm wide that extend from the leaf base to the apex (Fig. S2f). Lateral to each stomatal band is a narrow marginal zone. The stomatal bands are lighter than the median and marginal

zones of the leaf (Figs. 1b, S2f). Ratios of the widths of the midvein and stomatal band to the marginal zone are ca. 1.7: 2.1: 1.

Rectangular cell outlines preserved on impressions of both the adaxial and abaxial leaf surfaces are arranged in longitudinal files (Fig. S5a, S5b, S5c). Stomata are scattered in the stomatal bands with only three to four stomata across the width of the band (Fig. S5b, S5d). The guard cells are surrounded by six to eight polygonal to isodiametric subsidiary cells (Fig. S5d). Epidermal cells within the stomatal bands are non-papillate (Fig. S5d).

There are twelve seed-bearing structures that occur in a group near the apex of an ultimate shoot (Figs. 1a, S2b, S3a). Seed-bearing structures are oppositely arranged and borne individually in the axil of a normal vegetative leaf (Figs. 1g, S3b, S3c). Each seed-bearing structure consists of a short, naked axis 2.4–3.0 mm long, ca. 1.0 mm thick, that is completely free from its subtending leaf, and slightly incurved toward the axis of the shoot on which it is borne (Figs. 1g, 1h, S6a-S6e). Each axis bears a terminal swollen, spherical to ovoid structure 1.8–2.2 mm long, ca. 2.0 mm wide, which is composed of up to five pairs of tightly overlapping opposite and decussate bracts that enclose the presumed seed (Figs. 1h, S6a-S6e). The seed is not clearly visible and there is no prominent micropyle. Bracts are triangular to ovate in outline, up to ca. 2 mm long and 0.5–1.0 mm wide, with a more or less prominent keel on the abaxial surface (Figs. 1h, S6d, S6e). The bract apex is acute to rounded and the margin is entire (Figs. 1h, S6a-S6e). The abaxial surface of the bract has sparse stomata and rectangular to irregularly shaped epidermal cell outlines that are 80–95 μm long and 30–40 μm wide (Fig. S6f).

Comparison of the Daohugou fossils with fossil Taxaceae

Among the fossils of Taxaceae, three species known based on leafy shoot with attached seed-bearing structures are most informative. These are *Palaeotaxus rediviva* Nathorst from the Early Jurassic (Hettangian) of southern Sweden (Florin 1958), *Marskea jurassica* (Florin) Harris from the Middle Jurassic of Yorkshire, England (Florin 1958; Harris 1979) and *Taxus guyangensis* from the Early Cretaceous (Aptian-Albian) of Inner Mongolia, China (Xu et al. 2015). *Palaeotaxus rediviva* has seed-bearing structures singly arising from the axils of vegetative leaves and bearing one terminal, erect seed like those of the Daohugou fossils. However, in *P. rediviva*, the axes of the seed-bearing structures are covered by dense helically arranged scale leaves. *Palaeotaxus rediviva* also differs in having helically arranged leaves and helically arranged bracts subtending the terminal seeds. *Taxus guyangensis* differs from the Daohugou fossils in having helically arranged leaves, and seed-bearing structures that lack a distinct stalk. *Marskea jurassica* closely resembles the Daohugou fossils. It has decussately arranged leaves, uniovulate seed-bearing structures borne in the axil of a vegetative or scale leaf, and a terminal ovule subtended by pairs of decussate scales. However, *Marskea jurassica* differs from the Daohugou fossils in its smaller leaves (commonly 2.0 cm long and 2.0 mm wide) with papillae on the epidermal cells in the stomatal bands.

Taxaceae is also known based on detached seeds or detached axes of seed-bearing structures in the Jurassic and Cretaceous deposits. *Vesquia tournaisii* C.E. Bertrand from the Early Cretaceous Wealden Formation of Belgium is a lignitic seed-bearing structure with an attached, apparently mature, seed (1 cm in diameter) (Alvin 1960). The seed is much larger than the immature seeds of the Daohugou fossils, and *V. tournaisii* appears to combine features of several extant genera of Taxaceae (Alvin 1960). *Poteridion hallei* Harris from the Middle Jurassic of Yorkshire, England is a detached seed-bearing axis bearing dense scale leaves similar to extant *Taxus* (Harris 1979).

Characters and character states used in phylogenetic analysis

1. Phyllotaxis: helical (0); opposite (sub-opposite) (1). Mature leaves are helically inserted in *Taxus*, *Pseudotaxus* and *Austrotaxus*, but in opposite or sub-opposite pairs in *Amentotaxus*, *Torreya* and *Cephalotaxus*.
2. Leaf base strongly constricted: absent (0); present (1). In Taxaceae *sensu stricto* and *Cephalotaxus* leaf base is strongly constricted and stalk like, whereas in *Cunninghamia*, the leaf base is only slightly or hardly constricted.
3. Leaf margin: toothed (0); entire (1). In all extant species of Taxaceae *sensu stricto* and *Cephalotaxus* the leaf margin is entire, whereas in *Cunninghamia* the leaf margin is toothed.
4. Resin canals in leaves: present (0); absent (1). Resin canals are present in the leaves of *Amentotaxus*, *Torreya* and *Cephalotaxus*, but absent in those of the tribe Taxeae (Ghimire et al. 2014). The three Jurassic fossils, *Amentotaxus* from Daohugou, *Palaeotaxus rediviva*, *Marskea jurassica* are all based on compression fossils for which this feature is unknown, we therefore score them as (?).
5. Leaf length: < 4 cm (0); ≥ 4 cm (1). Leaves of extant *Amentotaxus* are typically much larger than those of most other extant Northern Hemisphere Taxaceae (Ferguson et al. 1978). We score the Daohugou fossils as (1) based on size of leaves (45–55 mm long) on the penultimate leafy shoot.
6. Midvein prominent on adaxial leaf surface: absent (0); present (1). A midvein that is inconspicuous on adaxial (upper) leaf surface is characteristic for extant *Torreya*. In extant *Amentotaxus*, *Cephalotaxus, Taxus*, *Pseudotaxus* and *Austrotaxus*, the midvein is prominent on adaxial leaf surface. We score *Palaeotaxus rediviva* and *Marskea jurassica* as (1) because they are both described as having a prominent midvein, which is also reflected in the adaxial leaf cuticle.
7. Stomatal complex: monocyclic (0); amphicylic (1). The type of stomatal complex is thought to be diagnostic among extant genera of Taxaceae (Elpe et al. 2017). In monocyclic stomata, the two guard cells are surrounded by a single ring of subsidiary cells, as in leaves of *Amentotaxus*, *Torreya* and *Pseudotaxus*. In amphicyclic stomata, the subsidiary cells are

further divided, resulting in a complete or incomplete encircling ring of cells, as in leaves of *Austrotaxus*, *Cephalotaxus* and *Taxus*. The cuticle of the Daohugou fossils is not well preserved, we therefore conservatively scored this feature as (?).

8. Papillae on epidermal cells in stomatal bands: absent (0); short and isodiametric (1); elongated (2). Leaves of extant *Taxus* have short and isodiametric papillae on the epidermal cells of the stomatal bands, while in leaves of extant *Torreya* these papillae are elongated. In extant *Amentotaxus*, *Pseudotaxus*, *Austrotaxus* and early diverging species of *Cephalotaxus* epidermal cells in the stomatal bands lack papillae (Elpe et al. 2017).
9. Seed-bearing structure: simple (0); compound (1). The seed-bearing structure of *Cephalotaxus* is interpreted as a compound cone, distinct from that of Taxaceae *sensu stricto*.
10. Position of seed-bearing structure: in the axil of a normal vegetative leaf (0); in the axil of a bud scale (1); terminal on a leafy shoot (2). *Cunninghamia* is scored as (2).
11. Axis of seed-bearing structure: entirely covered by dense scale leaves (0); predominantly naked and bearing several ovules (1); predominantly naked, with a terminal ovule (2). We consider the stalk of seed cone in *Cunninghamia* as homologous to the axis of the seed-bearing structure in Taxaceae and thus score *Cunninghamia* as (0): entirely covered by dense scale leaves.
12. Arrangement of bracts subtending the seed: helical (0); decussate (1). The arrangement of bracts subtending the seed is not always the same as the phyllotaxis of leaves. We score *Cunninghamia* as (?).
13. Aril: absent (0); enclosing only proximal part of seed (1); nearly completely enclosing seed except for extreme apex (2); completely enclosing seed (3). The extent of aril enclosure is diagnostic among extant genera of Taxaceae (Fu et al. 1999b; Farjon 2017).

Supplementary Table 1 Matrix of Character States for Taxa Analyzed.

Taxon	1	2	3	4	5	6	7	8	9	10	11	12	13
Cunninghamia konishii	0	0	0	0	0	0	0	0	0	2	0	?	0
Amentotaxus argotaenia	1	1	1	0	1	1	0	0	1	0&1	2	1	2
Torreya taxifolia	1	1	1	0	0	0	0	2	1	0	0	1	3
Pseudotaxus chienii	0	1	1	1	0	1	0	0	1	0	0	1	1
Austrotaxus spicata	0	1	1	1	1	1	1	0	1	1	0	0	2
Taxus cuspidata	0	1	1	1	0	1	1	1	1	0	0	1	1
Cephalotaxus oliveri	1	1	1	0	0	1	1	0	0	1	1	1	3
**Daohugou fossils***	1	1	1	?	1	1	?	0	1	0	2	1	?
Marskea jurassica*	1	1	1	?	0	1	0	1	1	0&1	2	1	2
Palaeotaxus rediviva*	0	1	1	?	0	1	1	0	1	0	0	0	2

Note. Unknown character states are indicated by question mark. Fossil taxa are highlighted in bold and with a star. Data based on Fu et al. (1999a, b), Eckenwalder (2009), Farjon (2017), Ghimire et al. (2014), Elpe et al. (2017), Florin (1958), Harris (1979).

References

Alvin KL 1960. On the Seed *Vesquia tournaisii* C. E. Bertrand, from the Belgian Wealden. *Ann Bot* **8**: 508–515.

Cheng Y, Nicolson RG and Tripp K *et al.* 2000. Phylogeny of Taxaceae and Cephalotaxaceae genera inferred from chloroplast matK gene and nuclear rDNA ITS region. *Mol Phylogenet Evol* **14**: 353–365.

Eckenwalder JE. 2009. Conifers of the world. Portland, USA: Timber Press.

Elpe C, Knopf P, and Stützel TH *et al.* 2017. Cuticle micromorphology and the evolution of characters in leaves of Taxaceae *s. l. Bot J Linn Soc* **184**: 503–517.

Farjon A. 2017. A handbook of the world's conifers, second, revised edition. Leiden-Boston: Brill.

Ferguson DK, Jähnichen H and Alvin KL. 1978. *Amentotaxus* Pilger from the European Tertiary. *Feddes Rep* **89**: 379–410.

Florin R. 1958. On Jurassic taxads and conifers from northwestern Europe and eastern Greenland. *Acta Horti Bergiani* **17**: 257–402.

Fu LK, Li N and Mill RR. 1999a. Cephalotaxaceae. In: Wu ZY and Raven PH (eds.), Flora of China, vol. 4. Beijing: Science Press, St. Louis: Missouri Botanical Garden Press, 85–88.

Fu LK, Li N and Mill RR. 1999b. Taxaceae. In: Wu ZY and Raven PH (eds.), Flora of China,

vol. 4. Beijing: Science Press, St. Louis: Missouri Botanical Garden Press, 89–96.

Ghimire B, Lee C and Heo K. 2014. Leaf anatomy and its implications for phylogenetic relationships in Taxaceae *s. l.* *J Plant Res* **127**: 373–388.

Harris TM. 1979. The Yorkshire Jurassic Flora, V. Coniferales. London: British Museum Natural History.

Huang D 2016. The Daohugou Biota. Shanghai: Shanghai Scientific & Technical Publishers, 2016 (in Chinese).

Leslie AB, Beulieu JM and Rai HS *et al.* 2012 Hemisphere scale differences in conifer evolutionary dynamics. *Proc Natl Acad Sci USA* **109**: 16217–16221.

Mao K, Milne RI and Zhang L *et al.* 2012. Distribution of living Cupressaceae reflects the breakup of Pangea. *Proc Natl Acad Sci USA* **109**: 7793–7798.

Na Y, Sun C and Wang H *et al.* 2017. A brief introduction to the Middle Jurassic Daohugou Flora from Inner Mongolia, China. *Rev Palaeobot Palynol* **247**: 53–67.

Pott C and Jiang B. 2017. Plant remains from the Middle-Late Jurassic Daohugou site of the Yanliao Biota in Inner Mongolia, China. *Acta Palaeobot* **57**: 185–222.

Ran J, Shen T, Wang M *et al.* 2018. Phylogenomics resolves the deep phylogeny of seed plants and indicates partial convergent or homoplastic evolution between Gnetales and angiosperms. *Proc R Soc B: Biol Sci* **285**: 20181012.

Shi G, Leslie AB and Herendeen PS *et al.* 2014. Whole-plant reconstruction and phylogenetic relationship of *Elatides zhoui* sp. nov. (Cupressaceae) from the Early Cretaceous of Mongolia. *Int J Plant Sci* **175**: 911–930.

Xu X, Sun B and Yan D *et al.* 2015. A *Taxus* leafy branch with attached ovules from Lower Cretaceous of Inner Mongolia, North China. *Cretaceous Res* **54**: 266–282.

Xu X, Zhou Z and Sullivan C *et al.* 2016. An Updated Review of the Middle-Late Jurassic Yanliao Biota: Chronology, Taphonomy, Paleontology and Paleoecology. *Acta Geol Sin* **90**: 2229–2243.
